# Neurosurgical treatment of gangliogliomas in children and adolescents: long-term follow-up of a single-institution series of 32 patients

**DOI:** 10.1007/s00701-018-3550-8

**Published:** 2018-04-22

**Authors:** Tryggve Lundar, Bernt Johan Due-Tønnessen, Radek Fric, Arild Egge, Bård Krossnes, Paulina Due-Tønnessen, Einar Stensvold, Petter Brandal

**Affiliations:** 1 Department of Neurosurgery, Oslo University Hospital, Postboks 4054, Nydalen, 0407 Oslo, Norway; 2 Faculty of Medicine, University of Oslo, Oslo, Norway; 3 Department of Pathology, Oslo University Hospital, Oslo, Norway; 4 Department of Radiology, Oslo University Hospital, Oslo, Norway; 5 Department of Pediatrics, Oslo University Hospital, Oslo, Norway; 6 Department of Oncology, Oslo University Hospital, Oslo, Norway

**Keywords:** Pediatric ganglioglioma, Long-term results, Pediatric neurosurgery, Oncology

## Abstract

**Object:**

The object of this study was to delineate long-term results of the surgical treatment of pediatric tumors classified as ganglioglioma or gangliocytoma.

**Methods:**

A cohort of consecutive patients 19 years or younger who had undergone primary resection of CNS tumors during the years 1980–2016 at a single institution were reviewed in this retrospective study of surgical morbidity, mortality, and academic achievement and/or work participation. Gross motor function and activities of daily living were scored using the Barthel Index (BI).

**Results:**

Patient records for 32 consecutive children and adolescents who had undergone resection for a ganglioglioma were included in this study. Of the 32 patients, 13 were in the first decade at the first surgery, whereas 19 were in the second decade. The male/female ratio was 1.0 (16/16). No patient was lost to follow-up. The tumor was localized to the supratentorial compartment in 26 patients, to the posterior fossa in 5 patients, and to the spinal cord in 1 patient. Only two of the tumors were classified as anaplastic. Of the 30 low-grade tumors, 2 were classified as gangliocytomas, 6 were desmoplastic infantile gangliogliomas, and 22 were ordinary gangliogliomas. The aim of primary surgery was gross-total resection (GTR) and was achieved in 23 patients (71.9%). Altogether, 43 tumor resections were performed. Eight patients underwent a second resection from 1 to 10 years after primary surgery and three of these also had a third resection from 2 to 24 years after initial surgery. The reason for further resection was clinical (seizure control failure/recurrence of epilepsy or progressive neurological deficit) and/or residual tumor progression/recurrence. There was no operative mortality in this series and all 32 patients are alive with follow-up periods from 0.5 to 36 years (median 14 years). Observed 14-year survival is thus 100%. One out of two children with primary anaplastic tumor received local radiotherapy (proton) postoperatively. The other 31 patients did not have any kind of non-surgical adjuvant therapy. Twenty-one out of 26 children with supratentorial tumor had epilepsy as one of their presenting symptoms. Nineteen of these became seizure-free after initial surgery (18 of them after GTR), but 3 patients experienced recurrence of seizures within some years. Functional outcome in terms of ADL, schooling, and work participation was gratifying in most patients. Five patients have persistent hydrocephalus (HC), treated with ventriculoperitoneal (VP) shunts.

**Conclusion:**

Low-grade gangliogliomas (GGs) can be surgically treated with good long-term results including seizure and tumor control as well as school and working participation.

## Introduction

Gangliogliomas are rare tumors occurring in children, adolescents, and adults. Due to their rarity, most clinical reports include a limited number of patients and long-term results are not known 1–3, 9, 10, 16, 21, 24–26]. A few larger single institutional series have been reported, but mostly include adults with a history of severe epilepsy for many years treated in centralized centers for surgical treatment of epilepsy [14]. This retrospective series report the long-term results of surgical treatment of pediatric gangliogliomas (GGs).

## Methods

We retrospectively analyzed a cohort of 40 consecutive patients 19 years or younger who had undergone primary resection of CNS tumors originally described as ganglioglioma or gangliocytoma during the years 1980–2016 in the Department of Neurosurgery at the National Hospital, Oslo, Norway. Cases were collected from surgical protocols of the given time period in which initial histological evaluation revealed ganglioglioma or gangliocytoma.

Recorded data included patient sex, age at the time of primary tumor resection, and management of hydrocephalus. We also noted scholastic outcome, which was simplified into normal versus special schooling, and employment, which was categorized as open (in the competitive labor market), sheltered (for handicapped individuals, often reduced time and financed by the social security system), or no work.

Computed tomography scans were used for tumor diagnosis and follow-up in the years 1980–1986. Since 1987, when MRI became available, the tumor was visualized on preoperative MR images, and repeat MRI was introduced in the follow-up. The aim of the surgical procedure was gross-total resection (GTR) or at least substantial tumor volume reduction. The degree of resection was evaluated on postoperative MRI, often while the patient remained under anesthesia from the primary resection.

The Barthel Index (BI) is a well-established and validated scale using 10 variables to measure performance in basic ADLs primarily related to personal care and mobility [15]. Scores range from 0 to 100, with a higher score denoting greater independence. We intended to assess functional status and illustrate eventual differences among subgroups within our cohort.

## Results

After a critical pathological review, 32 of the 40 tumors met updated and extended diagnostic criteria (WHO 2007): 2 gangliocytomas, 6 desmoplastic infantile gangliogliomas (DIG), and 24 gangliogliomas (2 of these were characterized as anaplastic and 22 low-grade). Some tumors were more difficult to classify than most, and international expert neuropathologists evaluated one of them. Nevertheless, this study includes all cases of ganglioglial pediatric gliomas surgically treated in this period, which represent 2.7% of all pediatric CNS tumors surgically treated at our institution in the same period.

### Tumor location and clinical presentation

In one patient, the tumor was localized to the spinal cord. This 14-year-old girl presented with paresis of the left leg and urinary incontinence (patient 6, Table 1).

**Table 1:** Clinical details of the 32 patients

Patient number	Sex/age	Location	Histology	Op year	Follow-up (years)	Present age (years)	Barthel Index	Resect grade
1	F/18	Supra	Ggl	1980	36	54	100	GTR
2	F/0.8	Supra	DIG	1980	36	37	100	GTR
3	F/8	Brainstem	Ggl	1980	36	45	100	GTR
4	M/7	Supra	Ggl	1983	33	40	100	GTR
5	F/4	Infra	Gglcyt	1989	27	31	100	STR
6	F/14	Conus	Ggl	1989	27	41	85	Biopsy
7	M/17	Supra	Ggl	1991	25	42	100	GTR
8	F/15	Supra	Ggl	1991	25	40	100	STR
9	M/7	Supra	Ggl	1994	22	29	100	STR
10	F/1	Infra	Ggl	1995	21	23	100	GTR
11	F/17	Supra	Ggl	1996	21	38	100	GTR
12	M/17	Supra	Ggl	1996	20	37	100	GTR
13	F/11	Supra	Ggl	1999	17	29	100	STR
14	M/8	Supra	Ggl	2000	16	25	100	STR
15	F/10	Supra	Ggl	2002	14	24	100	GTR
16	M/15	Supra	Gglanapl	2002	14	29	100	GTR
17	F/7	Supra	Ggl	2003	13	21	100	GTR
18	F/10	Supra	Ggl	2003	13	23	100	GTR
19	M/0.2	Supra	DIG	2004	12	13	100	STR
20	M/2	Supra	DIG	2007	9	12	100	GTR
21	M/0.4	Supra	DIG	2010	6	7	100	GTR
22	M/16	Supra	Gglcyt	2010	6	22	100	GTR
23	F/2	Supra	DIG	2010	6	8	100	GTR
24	F/11	Supra	Ggl	2011	5	16	100	GTR
25	F/13	Supra	Ggl	2011	5	18	100	GTR
26	M/18	Supra	Ggl	2012	4	23	100	GTR
27	M/11	Infra	DIG	2013	3	14	100	STR
28	M/12	Supra	Ggl	2013	3	16	100	GTR
29	F/16	Supra	Ggl	2014	2	19	100	GTR
30	M/9	Supra	Gglanapl	2015	1	11	100	GTR
31	M/18	Supra	Ggl	2015	1	19	100	GTR
32	M/11	Infra	Ggl	2016	0.5	12	100	GTR

Five patients had posterior fossa tumors (patients 3, 5, 10, 27, 32, Table 1). They presented with symptoms and signs of increased ICP, ataxia, nystagmus, and/or cranial nerve dysfunction.

Twenty-six children had supratentorial tumors and most presented with epilepsy (*n* = 21) and had hemispheric location. Some of these also had symptoms of elevated ICP (headache, papilledema) or rapidly increasing head circumference (patient 21). Two patients with a more axial tumor location (nucleus caudatus, thalamus) presented with hemiparesis as well as epilepsy.

Five of the 32 patients were also surgically treated (VP shunts) for hydrocephalus following tumor resection surgery. They had posterior fossa tumors (*n* = 3) or an “axial” (near midline) supratentorial tumor (*n* = 2).

Of the 21 patients presenting with seizures before the primary resection, 19 were initially seizure-free postoperatively, while 2 were not. In three patients where seizure control was initially successful, their epilepsy reappeared. Two of them became permanently seizure-free after a second resection and the last one after two repeat resections (patient 8).

On the other hand, two patients without preoperative epilepsy initially have experienced epileptic seizures after prolonged follow-up (after 5 and 21 years respectively), without MRI-proven tumor recurrence.

### Tumor location and extent of resection

According to radiological and preoperative findings, most tumors were unilateral and localized to the cerebral hemispheres: six frontal, eight temporal, three parietal, and two occipital.

**Fig. 1:**
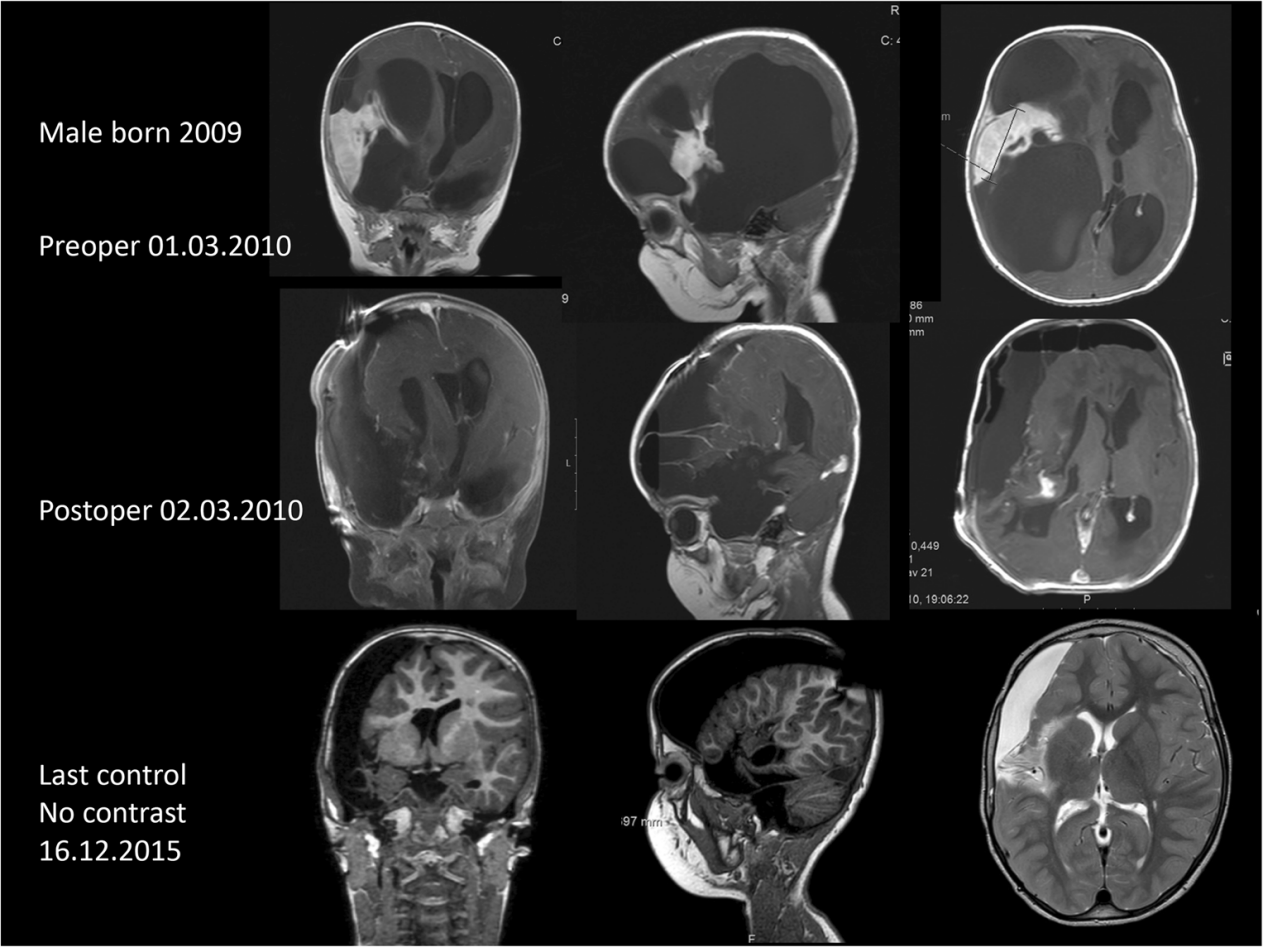
Pre- and postoperative as well as follow-up MRIs in patient 19

Four supratentorial tumors had mainly an intraventricular and/or more “axial” localizations (thalamus, nucleus caudatus, corpus striatum, trigonum). Furthermore, the last three supratentorial tumors were multilobar, including the Sylvian fissure or the whole hemisphere (Fig. 1).

All 32 patients underwent an open procedure with tumor resection whose aim was gross-total resection. In the early period of the study (patients 1–4), the degree of resection achieved was based on the surgeon’s description and/or CT scans. After its introduction in 1987, MRI better documented the extent of resection on immediate postoperative imaging. Gross-total resection was accomplished in 24 patients (Table 1), including patient 3, an 8-year-old girl who underwent GTR of a brainstem ganglioglioma localized to the pons/medulla oblongata in 1980. She presented with progressive hydrocephalus and cranial nerve dysfunction, has undergone two shunt revisions, but is tumor-free and in partial work after 36 years of follow-up. Seven patients underwent a subtotal resection during the primary operation (Table 1).

The only child who only had a small biopsy during the initial procedure was a 14-year-old girl who presented in 1989 with paresis of the left leg and urinary incontinence. MRI revealed a tumor in the conus which was not well delineated. A small biopsy demonstrated a low-grade ganglioglioma, and an external decompression including laminectomy and a dural patch was performed. A second procedure was undertaken in 1996 due to clinical as well as MRI-proven tumor progression. A subtotal resection was performed. However, she experienced clear progression of clinical symptoms, increased urinary incontinence, and partial encoprese. An increase in motor weakness has partially improved. Twenty years later (27-years of total follow-up), she has become a mother and is in partial work and with a Barthel Index of 85.

### Repeat surgery

In total, eight patients underwent a second resection (Table 2) after 0.5 to 10 years of follow-up. Three of these patients also had a third resection after further 1, 6, and 11 years of follow-up. The decision for further resection surgery was based on clinical progression and/or progression of MRI-based indications of increase of residual tumor extension (enhancement).

**Table 2:** Clinical details of patients with repeat surgery

Patient number	Sex/age	Location	Histology	Op year	Follow-up (years)	Present age (years)	Barthel Index	Resect grade
5	F/4	Infra	Gglcytoma	1989	27	31	100	STR
5	F/13	Infra	Gglcytoma	RE 1999	17	31	100	STR
6	F/14	Conus	Ggl	1989	27	41	85	Biopsy
6	F/21	Conus	Ggl	RE 1996	20	41	85	STR
8	F/15	Supra	Ggl	1991	25	40	100	STR
8	F/18	Supra	Ggl	RE 1994	22	40	100	STR
8	F/39	Supra	Ggl	RE 2015	1	40	100	GTR
9	M/7	Supra	Ggl	1994	22	29	100	STR
9	M/9	Supra	Ggl	RE 1996	20	29	100	STR
9	M/15	Supra	Ggl	RE 2002	14	29	100	STR
14	M/8	Supra	Ggl	2000	16	25	100	STR
14	M/10	Supra	Ggl	RE 2002	14	25	100	STR
16	M/15	Supra	Ggl anapl	2002	14	29	100	GTR
16	M/21	Supra	Ggl	RE 2009	7	29	100	GTR
24	F/11	Supra	Ggl	2011	5	16	100	GTR
24	F/14	Supra	Ggl	RE 2014	2	16	100	GTR
27	M/11	Infra	DIG	2013	3	14	100	STR
27	M/12	Infra	DIG	RE 2014	2	14	100	STR
27	M/13	Infra	DIG	RE 2015	1	14	100	STR

### Pathological analysis

Two patients were diagnosed with anaplastic (WHO grade III) tumors (patients 16 and 30). The first was a 15-year-old boy presenting with epilepsy in 2002. MRI showed an occipital, partially cystic tumor. After GTR, he was followed with annual pleasing MRI scans for 5 years.

Thereafter, a small but increasing residual tumor (with enhancement) showed up. A second resection was therefore undertaken. Further MRIs have since been uneventful, up to further 8 years of follow-up. He has not had epilepsy since the primary resection in 2002 and is in full work as an engineer 15 years after primary treatment. The second patient was a 9-year-old boy presenting with epilepsy in 2015. After GTR, he was diagnosed with anaplastic ganglioglioma. In spite of uneventful postoperative MRI, he was treated with postoperative radiotherapy (proton), according to international protocols. He is seizure-free and is doing quite well so far. This boy is the only one in this series of 32 children who has undergone non-surgical antitumor therapy.

The other 30 patients had low-grade tumors. Two of these were gangliocytomas, including a 4-year-old girl (patient 5) presenting in 1989 with posterior fossa symptoms (hydrocephalus, cranial nerve dysfunction). She was diagnosed with Lhermitte-Duclos disease (gangliocytoma of the posterior fossa). The tumor was localized to the left cerebellar hemisphere, but also in the part that invaded the brain stem. After STR, she was followed with annual MRI. Small residual tumor enhancements increased with time. After a good clinical period, she experienced clinical progression along with increased tumor size on MRI. She therefore underwent a second resection (STR) in 1999 at the age of 13 years.

**Fig. 2:**
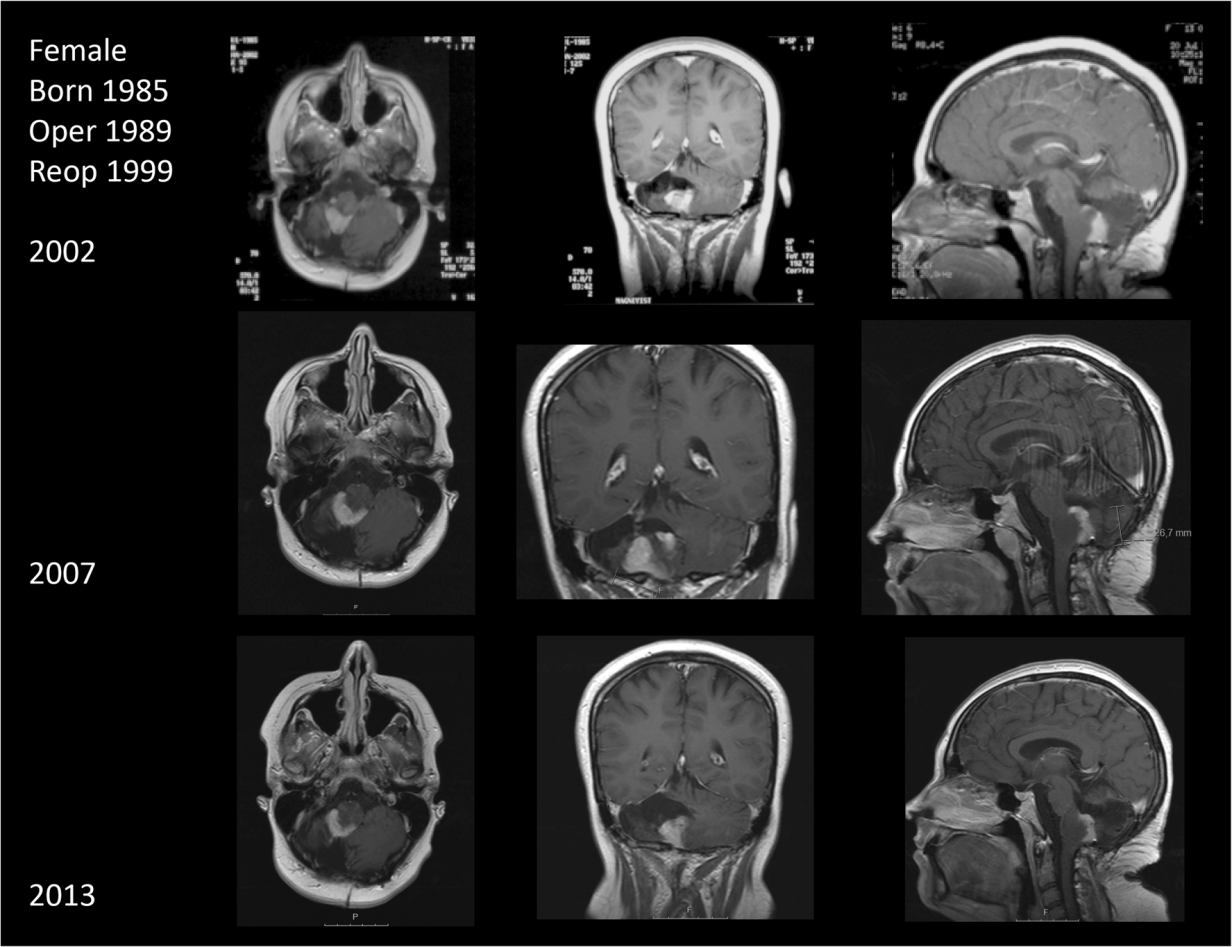
Follow-up MRIs in patient 5

Once more, MRI-proven residual tumor increased during some years. Her clinical condition has, however, been without progression for the next 18 years in spite of a substantial residual tumor (Fig. 2) without significant progression for the last 12 years. Her BI is 100, and she is in sheltered work and is performing horse riding.

### Desmoplastic infantile ganglioglioma

Six of the children with low-grade tumors were diagnosed as with desmoplastic infantile gangliogliomas (DIG). Three of these were in the first year of life (patients 2, 19, and 21) and two were 2 years old. They had aggressive and large tumors. The clinical presentation was epilepsy and/or signs of increasing ICP—increased head circumference, papilledema. The last child with DIG was a 13-year-old boy with typical symptoms and signs of a posterior fossa tumor—hydrocephalus, headache, and vomiting. The MRIs demonstrated a somewhat diffuse tumor, mostly in the left cerebellar complex. After STR in 2013, a second and a third resection (both STR) were undertaken in 2014 and 2015. He is doing well at school including basketball, and the MRIs appear stable (minor residual tumor manifestations).

### Mortality and survival

There was no operative mortality in this series and all 32 patients are alive. In 8 patients, repeat surgical resection was found indicated as described. The follow-up periods range from 0.5 to 36 years. The median follow-up was 14 years, and 12 patients have more than 20 years of follow-up since the first operative procedure.

### Postoperative function

Overall motor function and management of ADLs were good. In terms of the BI, the score was 100 in 31 patients and 85 in one patient.

Of the 32 patients, 5 have focal neurological deficits: residual hemiparesis in 1 child with hemiplegia preoperatively (patient 9—3 resections). He was hemiplegic all the time after resections of a thalamic tumor with recurrences.

Another one was a 15-year-old girl presenting with epilepsy in 1991 (patient 8). After STR, she was seizure-free for a couple of years, where after the fits reappeared. After a second resection in 1994, she once more was seizure-free for several years, but experienced a moderate paresis of her left foot, improving with time. Once more, her epilepsy reappeared along with MRI progression of residual tumor with close relation to the motor cortex. The third resection took place in 2015 after detailed MRI studies including tractography and fMRI: The procedure was performed with the patient awake, and a GTR was undertaken and confirmed with postoperative MRI. She experienced a partial paresis of the left foot with some improvement over time postoperatively. At present, she is once more seizure-free and hopefully she will remain tumor-free in the future. She is in 50% work in the open labor market.

The third patient with focal neurological deficit is the patient treated with resections of a conus tumor (patient 6). The two last patients with moderate neurological deficits are two children treated for posterior fossa tumors including the brain stem (patients 3 and 5). They have slight cranial nerve dysfunction but the overall motor function is good.

Currently, 12 of the 32 patients have an age below 20 years (7–19 years) and they all follow regular school programs except from one of the infants treated for a large left-sided hemispheric tumor (patient 19) with severe symptoms of autism. The remaining 20 patients are ages 20–54 years. Three are students, 14 are employed, and 3 are unemployed.

### Epilepsy

Twenty-one out of the 26 patients with supratentorial tumors presented with epilepsy before the primary resection. Nineteen of these children became initially seizure-free after surgery, but in two patients, the epilepsy reappeared. The other 17 patients became permanently seizure-free and in 16 of these, this was following a GTR procedure. Only 1 out of 5 patients became permanently seizure-free after a STR procedure.

Two of the 21 children with preoperative fits did not respond with initial seizure reduction. Both underwent repeat surgery and became seizure-free after the second resection after initial STR. They are, however, not completely tumor-free based on MRI evaluation, so the second resection has also been classified as STR (patients 9 and 14, Table 2). In patient 9, epilepsy reappeared. He underwent a third resection (Table 2) of a deep-seated thalamic lesion. The decision for further resection surgery was mostly based on tumor progression with increased hemiparesis and not on the epilepsy. He still has some fits, but they are well controlled with AED and he is now aged 29 and in partial work after another 14 years (out of total 22 years) of follow-up (Table 2).

The more complex details of patient 8 are described above. She is hopefully permanently seizure-free (but with only 1-year observation) after the third procedure, GTR 24 years after the first of two STR resections.

## Discussion

Gangliogliomas represent only a small proportion of gliomas in children and teenagers.

The present consecutive series covers a period of 37 years, in which remarkable diagnostic and operative progress have been made. This implies that over time, approximately one new case has been included annually. Our GG cases represent about 2.7% of the CNS tumors surgically treated in children and adolescents during the same period.

Some will claim that retrospective clinical studies like the present one are problematic since cases treated before the implementation of pre- and postoperative MRI are included. We fully agree that management in the 7 years before the MRI era was less precise, and that resection grade based on CT and the surgeons’ description is more uncertain. The present series is, however, relatively large and consecutive. Inclusion of patients over nearly four decades with complete follow-up gives us long-term follow-up data.

The present series of 32 consecutive cases diagnosed in children and adolescents between 1980 and 2016 confirms that ganglioglial tumors are mainly localized in the supratentorial compartment, most often in the hemispheres. Due to their rarity, single institutional series contain limited numbers of patients, and long-time follow-up results have not been published [20]. Dudley and coworkers published a series of 348 pediatric low-grade GGs treated during the years 2004–2010 based on the SEER data sets of the National Cancer Institute in the USA [4]. They found a male preponderance (60%) like in many other series, which was not seen in our series. Clinical presentation in the second decade and supratentorial tumor localization in our consecutive series were closely resembling the large SEER data sets. Since our series presents all pediatric ganglioglial tumors in the study period, it also presents small numbers of tumors with posterior fossa or spinal cord location. We agree that these tumors represent other challenges for the pediatric neurosurgeon than the larger group of mostly cortical supratentorial tumors. We feel that our limited experience with these six patients, nevertheless illustrates that they also could be managed over time by neurosurgical resection alone. Posterior fossa and spinal cord low-grade GGs are also included in the large SEER data sets. In contrast to the publication based on SEER data, we also included six children with desmoplastic infantile ganglioglioma (DIG) [2, 22]. Three of these were in the first year of life and may explain why 3 out of the 32 children in the present series were infants. Prognosis for children with DIG is considered to be more dismal than for children with low-grade GG. So far, the small number of children treated with resection alone and without radiotherapy appears promising.

Our experience confirms previous studies with good results with respect to seizure control in patients presenting with epilepsy, especially after GTR [14, 25].

Tomita and coworkers present a large pediatric series of patients with hemispheric glioneuronal tumors, including 58 low-grade GGs with excellent seizure and tumor control after surgical resection [25].

Several authors underline the importance of GTR at the primary surgical resection [6]. We achieved this in two out of three patients (71.9%). In the last nine children (28%), STR was performed in eight and a minimal biopsy in the last one. Any pediatric neurosurgeon will go for GTR, and favorable success rates with respect to persistent seizure control after GTR appear to be linked to this. Nevertheless, we saw lack of seizure control after GTR in 2 out of 21 patients and recurrence of epilepsy after successful GTR in one. The last phenomenon (initial success, but recurrence of epilepsy after a couple of years) was more common after STR. We did not see tumor recurrence after GTR in any child with low-grade tumor but in one out of two after GTR of grade III tumor (but without clinical symptoms).

The reason for not achieving GTR at primary surgery was tumor localization and risk of unacceptable postoperative neurological deficits as judged by the surgeon.

Two tumors were furthermore not well delineated on MRI, and therefore not candidates for radical surgery. We find it important to underline that the eight cases of initial STR procedures were also in our opinion partly successful, as the patients had “good outcomes” in terms of survival, ADLs, and function. Seven of the children underwent a repeat STR procedure, three of whom had a third resection (STR in two and GTR in one). These children generally tolerated further surgery well, and these experiences are the basis for our recommendation of repeat surgery instead of non-surgical adjuvant therapy.

Primary anaplastic gangliomas are rare [12]. Secondary malignant transformation in gangliomas has also been reported, but seems to be unusual [7, 23]. Several authors have discussed the use of postoperative radiotherapy for GG patients, especially after STR [11, 17]. The use of radiotherapy seems to have declined considerably during the last decades [4]. In fact, some authors claim that the risk for malignant development may actually increase after such treatment [18, 19].

In our limited experience with these 32 pediatric cases, we have not seen a single case with malignant development during the follow-up period in the low-grade cases.

The two cases with initial anaplastic tumor are for the time being tumor-free. The first one with repeat complete resection of recurrent tumor was 7 years after initial GTR, and thereafter 8 years uneventful survival in full work. The other one also underwent GTR but was given local proton therapy postoperatively according to protocol and has experienced an uneventful survival for 2 years. The harmful effects of modern radiotherapy are difficult to assess. Late detrimental consequences of such management are feared in children [8, 13].

Our series illustrate that low-grade GGs in children can be treated with rewarding long-term results based on surgery alone and without adjuvant radiotherapy even in cases with incomplete primary resection or recurrent disease. Repeat surgery should, in our opinion, be considered before radiotherapy is given in these children. The long-term results appear promising both with respect to survival, activities of daily living, school, and working ability in most of the patients. In patients presenting with epilepsy, good results were achieved when GTR could be performed. Tumor control (or stabilization of disease) was obtained also in cases with STR or recurrent disease with repeat surgery.

Hopefully, children with low-grade GGs may have a potential for a good adult life like we have seen in pediatric series of low-grade cerebellar astrocytomas [5].

## Conclusion

Pediatric ganglioglioma is a surgical disease with favorable long-term outcome in terms of survival, overall motor control, and ADL (Barthel Index). Clinical presentation was often first-time epileptic fits. Gross-total resection was achieved in 71.9% with good tumor and seizure control. Repeat surgery was, however, performed in 25% of the patients up to 24 years after initial surgery.

## Abbreviations

ADL: Activity of daily living
AED: Antiepileptic drug
BI: Barthel Index
GTR: Gross-total resection
STR: Subtotal resection
GG: Ganglioglioma
DIG: Desmoplastic infantile ganglioglioma
HC: Hydrocephalus
ICP: Intracranial pressure
VP: Ventriculoperitoneal
